# A Type VI Secretion System Is Involved in *Pseudomonas fluorescens* Bacterial Competition

**DOI:** 10.1371/journal.pone.0089411

**Published:** 2014-02-14

**Authors:** Victorien Decoin, Corinne Barbey, Dorian Bergeau, Xavier Latour, Marc G. J. Feuilloley, Nicole Orange, Annabelle Merieau

**Affiliations:** LMSM, Laboratoire de Microbiologie Signaux et Microenvironnement, Normandie Université, EA 4312, Université de Rouen, IUT d’Evreux, Evreux, France; Universidad Pública de Navarra, Spain

## Abstract

Protein secretion systems are crucial mediators of bacterial interactions with other organisms. Among them, the type VI secretion system (T6SS) is widespread in Gram-negative bacteria and appears to inject toxins into competitor bacteria and/or eukaryotic cells. Major human pathogens, such as *Vibrio cholerae*, *Burkholderia* and *Pseudomonas aeruginosa,* express T6SSs. Bacteria prevent self-intoxication by their own T6SS toxins by producing immunity proteins, which interact with the cognate toxins. We describe here an environmental *P. fluorescens* strain, MFE01, displaying an uncommon oversecretion of Hcp (hemolysin-coregulated protein) and VgrG (valine-glycine repeat protein G) into the culture medium. These proteins are characteristic components of a functional T6SS. The aim of this study was to attribute a role to this energy-consuming overexpression of the T6SS. The genome of MFE01 contains at least two *hcp* genes (*hcp*1 and *hcp*2), suggesting that there may be two putative T6SS clusters. Phenotypic studies have shown that MFE01 is avirulent against various eukaryotic cell models (amebas, plant or animal cell models), but has antibacterial activity against a wide range of competitor bacteria, including rhizobacteria and clinical bacteria. Depending on the prey cell, mutagenesis of the *hcp*2 gene in MFE01 abolishes or reduces this antibacterial killing activity. Moreover, the introduction of T6SS immunity proteins from *S. marcescens*, which is not killed by MFE01, protects *E. coli* against MFE01 killing. These findings suggest that the protein encoded by *hcp2* is involved in the killing activity of MFE01 mediated by effectors of the T6SS targeting the peptidoglycan of Gram-negative bacteria. Our results indicate that MFE01 can protect potato tubers against *Pectobacterium atrosepticum*, which causes tuber soft rot. *Pseudomonas fluorescens* is often described as a major PGPR (plant growth-promoting rhizobacterium), and our results suggest that there may be a connection between the T6SS and the PGPR properties of this bacterium.

## Introduction

Bacteria have developed an arsenal of mechanisms, including secretion systems to enable them to resist the various stresses generated by their environment. Secretion systems are critical weapons allowing bacteria to persist in an ecological niche or to conquer new one. The most recently described secretion system in the Gram-negative *Proteobacteriaceae* is the type VI secretion system (T6SS) [1], [2]. This protein complex releases virulence factors into the extracellular medium or transports them directly into the target cell. T6SS is a macromolecular machine involved in bacterial virulence and/or interactions with other organisms [3]. T6SS gene clusters differ between bacterial species in terms of gene order and orientation, but all have a conserved group of 13 essential genes, the “core components” [4]–[6]. Two of the core genes, *hcp* and *vgrG*, encode the extracellular part of the secretion machinery. The Hcp and VgrG proteins are released into the culture medium by T6SS activity. This release provides evidence that the T6SS apparatus is functional [7]. The T6SS seems to consist of a needle-like membrane-puncturing device similar to a bacteriophage tail. Hcp and VgrG have secondary structures similar to those of bacteriophage components, with the major phage tail protein and the tail spike phage protein corresponding to Hcp and VgrG, respectively [8], [9]. The T6SS is implicated in virulence in some human pathogens, including *Vibrio cholerae*, *Burkholderia pseudomallei*, *Aeromonas hydrophila*, *Acinetobacter baumannii*, and *Pseudomonas aeruginosa* [6], [10]–[15]. However, the contribution of the T6SS to virulence remains unclear.

Several studies have reported that some T6SSs are used to kill competing bacteria [16]–[18]. Some T6SS toxins or effectors with a peptidoglycan hydrolase structure [19] are responsible for this killing activity. English and coworkers have reported the identification and characterization of two *S. marcescens* effectors [20] Ssp1 and Ssp2, which have this peptidoglycan hydrolase-like structure [21]. Phospholipase antibacterial effectors were also described [22] and other effectors of unknown activity were identified [17], [23], [24]. The self-intoxication of bacteria by their own toxins is prevented by the production of immunity proteins, which must interact physically with cognate toxins. Such interactions have been demonstrated in *P. aeruginosa* (Tsi proteins) and *S. marcescens* (Rap proteins) [20], [25]. The bacteria must come into close contact with prey cells for the T6SS to delivery toxins and exert its antibacterial activity [14], [26].

T6SSs are also widespread in many Gram-negative environmental bacteria. Many *Pseudomonas fluorescens* strains have genes encoding T6SS components [27]–[29]. These genomic and transcriptomic studies have suggested that the T6SS may be involved in interactions of *Pseudomonas fluorescens* with plants.

In this study, we identified an Hcp protein as the major supernatant protein of an environmental strain of *Pseudomonas fluorescens*, MFE01. This indicates that a T6SS of this strain was functional and constitutively overexpressed. We therefore investigated the role of this T6SS overexpression, by phenotypic and genetic studies.

## Materials and Methods

### Bacterial Strains, Plasmids and Culture Conditions

All strains and plasmids are listed in **Table 1**. All bacterial strains were grown in LB medium with shaking (180 rpm), except for *Pectobacterium atrosepticum* CFBP 6276, which was grown in PGA minimal medium supplemented with polygalacturonic acid 0.4% (wt/vol) (Sigma-Aldrich, St. Louis). *Pseudomonas fluorescens* strains were grown at 28°C or 37°C, *Pseudomonas aeruginosa* and *E. coli* at 37°C, and *Pectobacterium atrosepticum* was grown at 25°C [30]. When required, media were supplemented with antibiotics: kanamycin (Km), 50 µg/ml (*E. coli* or conjugation) or 100 µg/ml (*P. fluorescens* strains); tetracycline (Tc), 15 µg/ml; rifampicin (Rif), 25 µg/ml.

**Table 1:** Plasmids and strains included in the study.

Strains or plasmids	Relevant characteristics	Reference/source
Strains		
*P. fluorescens*		
MFE01	Air isolate, Rif^R^	This study
MFE01Δ*hcp2*	MFE01 with early stop codon in *hcp2*	This study
MFE01Δ*hcp2*.R	MFE01Δ*hcp2* revertant with wild-type *hcp2* gene in its original chromosomal location	This study
MFE01Δ*hcp2/hcp2*	MFE01Δ*hcp2* with pPSV35 carrying the wild-type *hcp2* gene	This study
MFN1032	Clinical strain able to grow at 37°C	[35]
MFP05	Skin isolate	LMSM collection
Pf01	Soil isolate	[43]
*P.aeruginosa*		
H103	Prototrophic derivative of PAO1	[37]
PA14	Clinical isolate	[32]
*Escherichia coli*		
K12		LMSM collection
DH5αmcr	General cloning strain	Bethesda Research Laboratories
S17.1	*RP4-2-Tc ::Mu aph ::Tn7 recA,* Sm^R^ donor strain for conjugation	[42]
*Pectobacterium atrosepticum* 6276	Isolate from *Solanum tuberosum*	[30]
*Serratia marcescens*		LMSM collection
*Klebsiella aerogenes*		[46]
Vectors		
pME6000	Replicative plasmid in *Pectobacterium atrosepticum* Tc^R^	[34]
pSMC21	Replicative in Gram-negative bacteria Km^R^, *gfp*	[33]
pAKE604	Conjugative suicide vector for *hcp2* gene mutagenesis; Km^R,^ *sac*B	[41]
pSUPROM	Km^R^, vector for constitutive expression of *rap* (*1a, 1b, 2a* or *2b*) genes under control of *E. coli tat* promoter	[20]
pPSV35	*P. aeruginosa oriV, lacIq mob+ PlacUV5*, pUC18 MCS, expression vector, Gm^R^	[49]

### Growth Curve and Hcp Secretion Analysis

*P. fluorescens* strains were cultivated in 25 ml of Luria Bertani medium in a 250 ml Erlenmeyer flask, with shaking at 180 rpm from an OD_580_ of 0.06. OD_580_ was measured at 45-minute intervals over a nine-hour period. Hcp secretion was assessed by harvesting the supernatants by centrifuging the cultures at 5000× *g* for 10 minutes at 20°C and passing them through a Millipore membrane with 0.22 µm pores. TCA was added to the supernatant to a final concentration of 10% and the mixture was incubated overnight at 4°C. The supernatant was removed by centrifugation at 13000× *g*, for 30 minutes at 4°C. The protein pellet was washed twice with 5 ml of 20 mM Tris base in cold acetone and centrifuged at 13000× *g*, for 30 minutes at 4°C. The dry pellet was then resuspended in distilled water. Proteins were separated by sodium dodecyl sulfate polyacrylamide gel electrophoresis (SDS-PAGE). Briefly, samples were mixed with an equal volume of 2× Laemmli sample buffer (with β-mercaptoethanol), boiled for 5 min at 100°C and then cooled to room temperature before loading.

### Mass spectrometry analysis

Mass spectroscopy (MS) analyses were performed in positive ion mode as described by Barbey *et al.* [31]. Statistical analyses of the sequences were carried out by determining the probability-based on Mowse score with MASCOT software (peptide tolerance = 100 ppm and mass values = MH+). A p-value of less than 0.05 was considered significant. The criteria used to accept protein identification based on peptide mass fingerprinting (PMF) data included the score probability greater than the score threshold 84 (p<0.05), the extent of sequence coverage (minimum 30%) and the number of matched peptides (minimum 8).

### Amplification of MFE01 T6SS Genes and Bioinformatics Analysis

For the amplification of Pf01-like *hcp* genes from *P. fluorescens* MFE01, we used the oligonucleotide primers Pfl01_2045F and Pfl01_2045R, and Pfl01_2328F and Pfl01_2328R (Table 2), designed on the basis of the genomic sequence of Pf01, for standard PCR with *P. fluorescens* MFE01 genomic DNA. The PCR parameters used were as follows: an annealing temperature of 56°C, an extension time of 25 s and 25 cycles. The polymerase used was the High Fidelity PCR Enzyme (Thermo Scientific). PCR fragments were inserted into the pGEM®-T-easy vector (Promega) and sequenced. Bioinformatics analyses were performed with Blast (NCBI web site).

**Table 2:** Oligonucleotides used for this study.

Primer name	Primer sequence (5′-3′)
Pfl01_2045F	ACCGGCGAAAAACAAGGCCTGA
Pfl01_2045R	ACGCCAGTCATCGGAACCCG
Pfl01_2328F	ATCACTGCCGGCGCGTTCA
Pfl01_2328R	GACCGGAGCACGCCAGTCATC
muta1hcp	CCTGGCGACTTTCTCCCGGT
muta2hcp	ATCGGAACCCGAAGTACCGGATT ATACTTCGTGGGTCCAGGTGAT
muta3hcp	ATCACCTGGACCCACGAAGTATAA TCCGGTACTTCGGGTTCCGAT
muta4hcp	TCGTAGGCGTTGCTCTGGATGC
hcp-vgrgF	ATCACCTGGACCCACGAAGT
hcp-vgrgR	CCCTTGTTCCTCGTGCTGTA
vgrGqpcrF	TTTACGCCCCTCCAGAATTT
vgrGqpcrR	AAAGACAAAAGGCTGGCTGAT
16S F	CTGGTAGTCCACGCCGTAAAC
16S R	CCAGGCGGTCAACTTAATGC

### Antibacterial Competition Assay

Antibacterial competition assays were carried out with a slightly modified version of a protocol described elsewhere [26]. To ensure selection of competitor cells for counting, *E. coli* K12, *P. aeruginosa* PA14 [32] and *P. fluorescens* strains (except MFE01 or MFE01Δ*hcp2*) were transformed with pSMC21 [33] to confer kanamycin resistance and *Pectobacterium atrosepticum* was transformed with pME6000 to confer tetracycline resistance [34]. *S. marcescens* was selected by plating on Hektoen medium plates without prior manipulation. The bacterial cells were grown overnight on LB agar plates and resuspended in LB. The OD_580_ was adjusted to 0.5, and the cells were mixed at a ratio of 5∶1, *P. fluorescens* MFE01: target bacteria. 25 µl of this mixture were spotted onto a filter with 0.22 µm pores on a prewarmed LB agar plate, which was then incubated for 4 h at 28°C or 37°C. The bacteria on the filter were resuspended in 1 ml of sterile physiological water and serial dilutions were plated on LB agar supplemented with antibiotic or selective medium. In contact-dependent competition experiments between *P. fluorescens* MFN1032 [35] and MFE01, the strains were separated by a filter with 0.22 µm pores. 25 µl of a suspension of one strain were spotted onto a filter, which was placed on the surface of the LB agar. A second filter with 0.22 µm pores was placed on top of this spot and 25 µl of the other strain was spotted onto this second filter.

### Cytotoxic Assay on Rat Glial Cells

We investigated the cytotoxic activity of *P. fluorescens* MFE01 with primary cultures of rat glial cells, as previously described [36]. Briefly, rat glial cells, obtained from newborn (24–48 h) rat brain, were grown in DMEM/Ham’s medium (2/1) supplemented with 10% fetal calf serum, 2 mM glutamine, 0.001% insulin, 5 mM HEPES, 0.3% glucose and 1% antibiotic-antimycotic solution (Biowhittaker, Emerainville, France). The cells were layered, at a concentration of 10^5^ cells/well, on 24-well plates coated with poly-L-lysine (50 µg.ml^−1^) and incubated at 37°C under a humidified atmosphere containing 5% CO_2_. Glial cells were allowed to grow for 12 to 16 days before use. Stationary-phase MFE01 cells were harvested by centrifugation at 8000× *g* for 5 minutes at room temperature. The bacteria were then resuspended at a density of 10^6^ cfu/ml in glial cell culture medium without antibiotics or antimycotics and incubated with the glial cells for 24 h. The concentration of LDH (a marker of necrosis) released by rat glial cells was determined with the Cytotox 96® Enzymatic Assay (Promega, Charbonnieres, France).

### Cytotoxicity Assay on Chicory Leaf

Strains were grown overnight, at 28°C for MFE01 or 37°C for *P. aeruginosa* H103 [37], with shaking (180 rpm). 1 ml of culture was centrifuged in a benchtop centrifuge and the pellet was resuspended in 1 ml of sterile 10 mM MgSO_4_. 10 µl of a suspension with an OD_580_ of 0.1 were injected into the central vein of a chicory leaf, which was then incubated for 24 h at 28°C or 37°C. Soft rot manifested as the appearance of a brown area around the injection.

### Soft-rot Test in Potato Tubers

*Pectobacterium atrosepticum* 6276 was prepared from stationary-phase cultures grown in PGA minimal medium supplemented with polygalacturonic acid 0.4% (wt/vol) (Sigma-Aldrich, St. Louis). Culture of *Pectobacterium* was centrifuged and the pellet was resuspended in sterile physiological water to obtain a suspension of 10^9^ cfu/ml. *P. fluorescens* MFE01 was suspended in 1 ml of sterile water, and the OD_580_ was adjusted to 1. Then *P. fluorescens* MFE01 or MFE01Δhcp2 were mixed with *Pectobacterium* at a ratio of 10∶1. *Solanum tuberosum* cv. Allians tubers were surface-sterilized and infected by the intramedullary injection (at a depth of 1 cm) with 10 µl of the bacterial mix. The inoculated tubers were incubated in a Minitron incubator (Infors, Massy, France) at 25°C and a relative humidity of 65% ±2%. For each assay, we analyzed 10 tubers, by noting the development of symptoms, by measurements of the diameter of soft rot, seven days after inoculation.

### *Dictyostelium discoideum* Growth and Predation Assays

This assay was performed exactly as described by Sperandio *et al.* [38].

### RNA Extraction, qRT-PCR and Co-transcription Assay

RNA was extracted by a modified version of the phenol-based extraction procedure described by Crépin *et al.* [39]. The cells were first lysed with the following lysis buffer: 0.02 M sodium acetate, pH 5.5, 0.5% (w/v) SDS, 1 mM EDTA. The cell lysates obtained were subjected to two consecutive phenol extractions, followed by a chloroform extraction. Total RNA was precipitated in 100% ethanol (2∶1, v/v) and 1 M sodium acetate (1∶10, v/v) and resuspended in RNase-free water. DNase treatment with RNAse-free Ambion® TURBO™ DNase (Life Technologies™) was carried out to remove any contaminating DNA. The quality and concentration of RNA samples were checked by agarose gel electrophoresis and with a Nanodrop spectrophotometer (Bio-Rad Laboratories). The absence of genomic DNA contamination was confirmed by PCR. RT-PCR was performed with 50 ng of RNA as a template, with the Transcriptor one-step RT-PCR kit (Roche, Meylan, France), according to the manufacturer’s recommendations. For cotranscription assays, we used the hcp-vgrgF and hcp-vgrgR primers (Table 2) for PCR assays. PCR was carried out in standard conditions, as follows: annealing temperature, 57°C; extension time, 15 s; 25 cycles; polymerase: Phusion® High-Fidelity DNA polymerase (NEB). mRNAs of *vgrG* were quantified by real-time PCR as described by Guyard-Nicodème and coworkers with minor modifications [40]. Primers (Table 2) were designed with Primer express 3 software and validated by PCR. PCR reactions were performed with the 7500 Fast real-time PCR system (Applied Biosystems). The 13 *µ*L reactions contained 6,5 *µ*L of SYBR Green PCR Master Mix (including AmpliTaq Gold DNA Polymerase, Applied Biosystems), 0,2 µM final of each primer and 13 ng final of cDNAs. PCR conditions as follows: 95°C for 20 sec, 40 cycles at 95°C, 57°C and 72°C for 3 sec, 30 sec and 15 sec respectively, 95°C for 15 sec, 60°C for 1 min and 95°C for 15 sec. The relative quantification of the mRNAs was obtained by the comparative CT (2^–ΔΔCT^) method as described [40].

### Construction of the *hcp2* Mutant of *P. fluorescens* MFE01

A 1.3 kb fragment containing the *hcp2* gene, inserted into pUC19, was obtained from an MFE01 genomic bank. A marker-less *hcp2* mutant was constructed by overlap PCR mutagenesis and the conjugative suicide vector pAKE604 [41] was selected on the basis of kanamycin resistance and sucrose sensitivity (Table 1). An early stop codon (TAA) was introduced in the middle of the *hcp2* gene. This was achieved by PCR with the muta1hcp and muta2hcp (≈700 bp product) or muta3hcp and muta4hcp (≈600 bp product) (Table 2). The PCR products obtained corresponded to the upstream and downstream parts, respectively, of the 1.3 kb genomic MFE01 fragment, each carrying an overlapping sequence at the end including the early stop codon. PCR parameters were as follows: annealing temperature, 53°C; extension time, 35 s; 30 cycles. A third PCR was then carried out in which the overlapping sequences of two first products were hybridized together and the product of polymerization with the muta1hcp and muta4hcp primers was a mutated *hcp2* gene. The PCR parameters were as follows: annealing temperature, 53°C; extension time, 35 s and 30 cycles. Each PCR was performed in standard conditions, with the Phusion® High-Fidelity DNA polymerase (NEB). The PCR fragment obtained was inserted into pAKE604 that had been linearized by digestion with *Sma*I (NEB). The resulting plasmid, pAKE604Δ*hcp2,* was verified by sequencing and was then transferred into MFE01 by biparental mating. *E. coli* S17-1 [42] containing pAKE604Δ*hcp2* and recipient MFE01 cells were mixed and spotted onto sterile nitrocellulose filters, which were placed on LB agar plates and incubated overnight at 37°C. The mating mixture was suspended in 1 ml of sterile physiological water and 0.1 ml aliquots were spread on LB agar plates supplemented with kanamycin (50 µg/ml), to select for the presence of the integrated plasmid, and rifampicin (25 µg/ml), to kill the *E. coli* S17-1 donor bacteria and to ensure the selection of MFE01. The excision of the suicide plasmid, by a second recombination event, was triggered by plating on LB agar medium supplemented with 10% sucrose. The mutant containing the early stop codon in the *hcp2* gene was verified by DNA sequencing and named MFE01Δ*hcp2.*

### Statistical Analysis

Data were analyzed using non-parametric Mann-Whitney Test (two tailed) with GraphPad Prism version 6.0 (La Jolla, CA). A *p-value* <0,05 was considered to be statistically significant.

## Results and Discussion

### *P. fluorescens* MFE01 Secretes Hcp-like Protein and VgrG-like Protein

**Figure 1:**
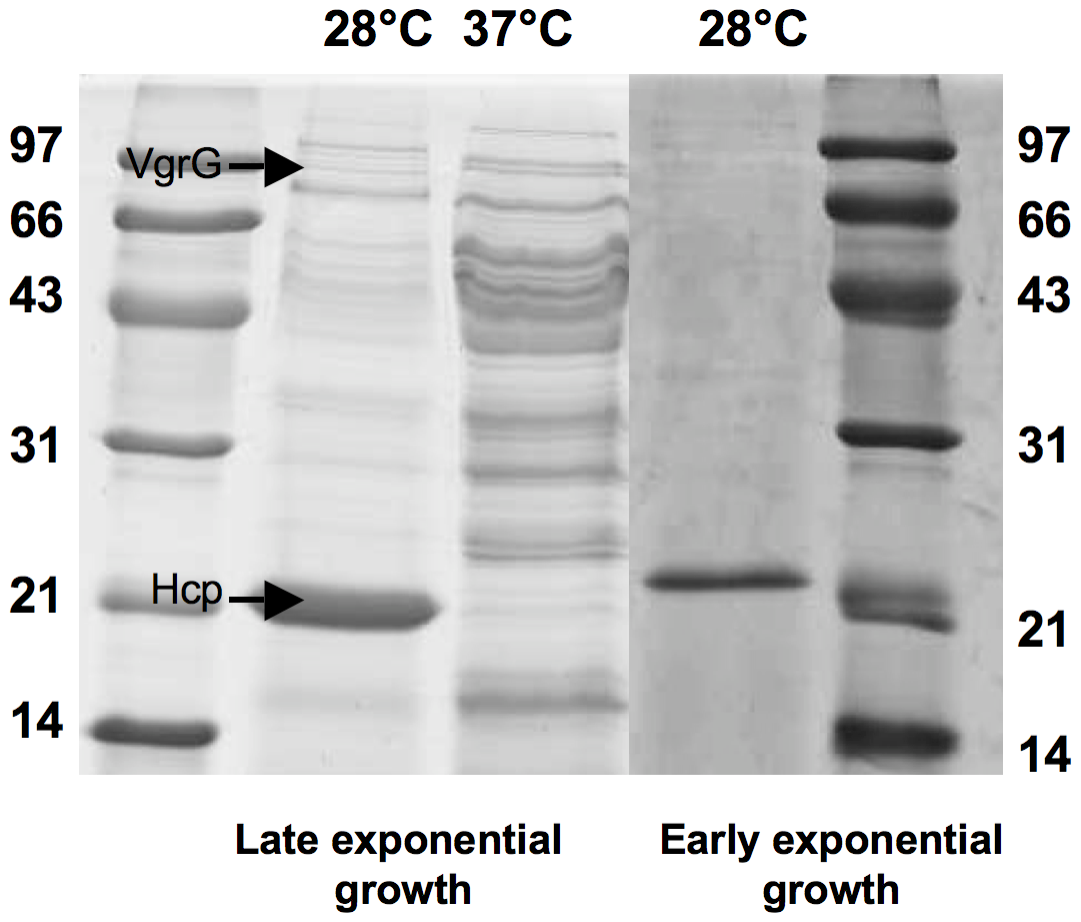
*Pseudomonas fluorescens* strain MFE01 secretes Hcp-like and VgrG-like proteins. Concentrated supernatants of MFE01 cultures were analyzed by SDS-PAGE and Coomassie Blue staining. A band with an approximate molecular mass similar to that expected for an Hcp protein (≈21 kDa), indicated by an arrow, was observed in the early exponential growth phase and late exponential growth phase of cells grown at 28°C but not at 37°C. Mass spectrometry identified this major supernatant protein as an Hcp-like protein. The second arrow indicates a VgrG-like protein from cells in late exponential growth phase at 28°C, absent at 37°C, with an approximate molecular mass of ≈85 kDa (identification by mass spectrometry).

MFE01 is an environmental bacterium with an optimal growth temperature of 30°C that can growth at 37°C. The proteins present in the culture supernatant were precipitated during the early exponential growth phase at 28°C and 37°C. A 20 kDa band was clearly visible after growth at 28°C, but not after growth at 37°C (Fig. 1). This protein was identified by mass spectrometry as an Hcp (hemolysin coregulated protein)-like protein. It matched with YP_348060 (type 6 secretion system effector, hcp1 family from *P.fluorescens*) with a score of 113, coverage of 48% and a number of peptides of 8.

During the late stationary growth phase at 28°C, an 85 kDa band, absent at 37°C, was identified by mass spectrometry as a VgrG-like protein (Fig. 1). It matched with WP_003225156 (type VI secretion protein Rhs from *P. fluorescens*) with a score of 103, coverage of 38% and a number of peptides of 23.

The secretion of Hcp and VgrG into the medium has been described as the “hallmark” of a functional T6SS [7]. The putative Hcp is the major protein present in the supernatant, from the early exponential growth phase onwards, suggesting an unusual pattern of constitutive T6SS expression at 28°C. We extended our analysis of Hcp secretion to other *P. fluorescens* strains. Pf01 secreted Hcp, but in smaller amounts. MFP05, an human skin isolate, and MFN1032, a clinical strain, did not secrete Hcp into the supernatant (data not shown). Thus, this oversecretion of Hcp does not appear to be a common feature of the species *Pseudomonas fluorescens*.

A growth temperature of 37°C corresponds to the regulatory “shutoff” temperature for the T6SS. This “shutoff” temperature has not been definitively determined. This temperature dependence suggests that MFE01 may use T6SS in the environment, but not in association with homoeothermic organisms.

### MFE01 has at Least Two *hcp*-like Genes

PCR primers designed on the basis of the sequence of Pf01 *hcp* genes [43] were used for the amplification of MFE01 chromosomal DNA (Table 2). Two amplicons, corresponding to the Pfl01_2328 (462 bp) and Pfl01_2045 (474 bp) primers, were obtained. These two partial sequences were checked by sequencing and subjected to Blast analysis. The gene *hcp1 corresponding to* the Pfl01_2328 primers displayed 95% identity to *hcp* Pfl01_2328 (partial sequence *hcp*1 genBank accession number: KF447439), whereas the gene *hcp2 corresponding to* the Pfl01_2328 primers displayed 97% identity to *hcp* Pfl01_2045 (complete sequence *hcp2* genBank accession number: KF447438). Partial *hcp1* gene sequence (462 bp) displayed 99% identity with *hcp2* gene (516 bp) with a query cover of 89%. Barret and coworkers have suggested that there is a relationship between the *P. aeruginosa* T6SS locus and VgrGs/Hcps sequences [29], arguing that the *P. aeruginosa* HSI-II could be linked to virulence in animals and plants, whereas HSI-I mediates interbacterial interactions [44]. Since, HSI-II also has been demonstrated to mediate interbacterial interactions [22]. However, the phylogenetic clusters probably evolved to adapt to various environments, so it is difficult to find a correlation between *hcp* and *vgr*G sequences, T6SS clusters and ecological niches [10]. We therefore decided to explore the virulence and interbacterial interactions of MFE01, to determine the role of the T6SS in this strain.

### *P. fluorescens* MFE01 Displays Antibacterial Activity Rather Than Eukaryotic Virulence

**Figure 2:**
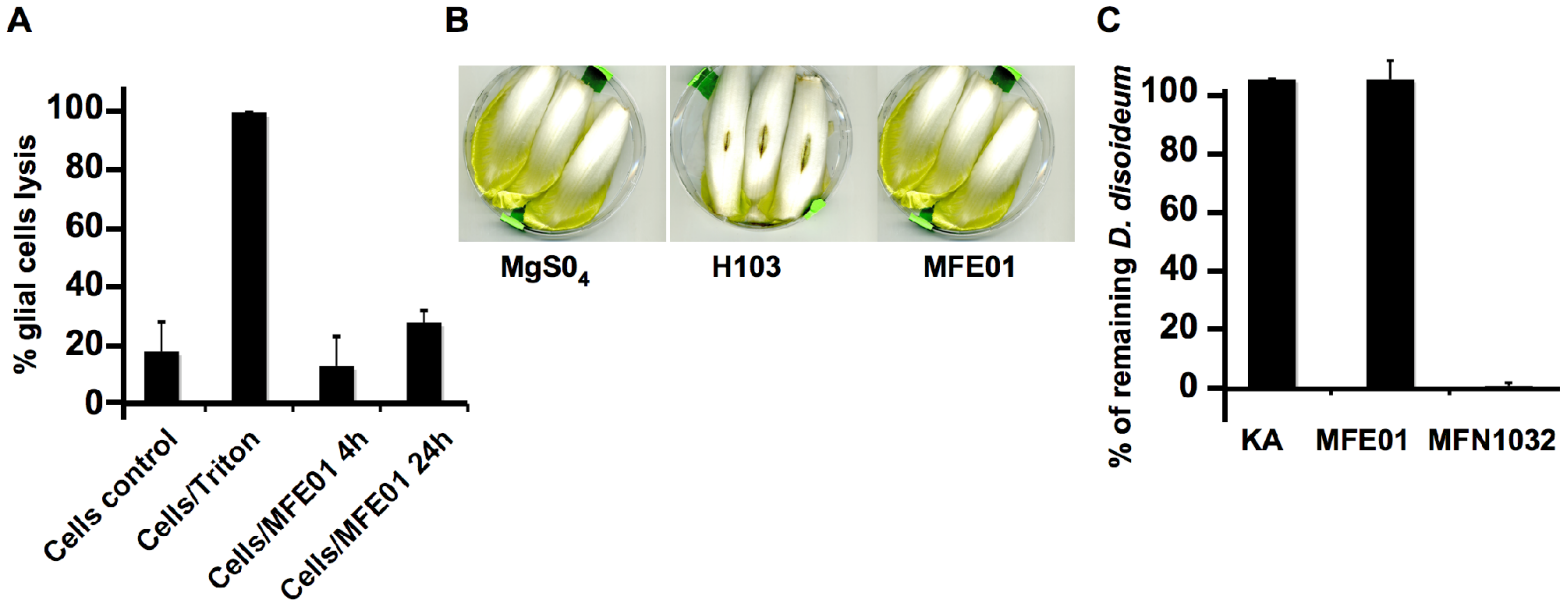
*P. fluorescens* MFE01 cytotoxicity toward amebas and animal and plant cells. (**A**) Lactate dehydrogenase release was used as a marker of cell lysis. Wild-type MFE01 and glial cells were incubated together, at 37°C, for 4 h and 24 h (*N* = 3). Error bars indicate the standard error of the mean. The control cells were used to determine the basal rate of lysis of the glial cell culture (Cells control). Cells were incubated with Triton to obtain 100% lysis as a positive control (Cell/triton). Cells/MFE01 corresponds to contact between glial cells and the MFE01 strain for the period indicated (4 h or 24 h). (**B**) The chicory leaf soft-rot assay was performed with wild-type MFE01 at 28°C (*N* = 3). “MgSO_4_” indicates a representative photograph of the outcome of injecting MgSO_4_ into the central vein and is a negative control for chicory leaf soft rot. “H103” shows a representative result for the inoculation of *P. aeruginosa* strain H103 and servers as a positive control for chicory leaf soft rot. “MFE01” shows a representative photograph of the effect on inoculation with *P. fluorescens* MFE01. (**C**) Approximately 100 *D. discoideum* AX3 cells were cultured on SM-plates, on a layer of *Klebsiella aerogenes* with or without 10% *Pseudomonas fluorescens* MFE01 or MFN1032. Plates were maintained at 22°C for 5 days. Lysis plaques were counted and compared with those obtained for the negative control for virulence, *Klebsiella aerogenes* (KA:100% of the amebas remain) and the positive control for virulence, *P. fluorescens* MFN1032 (0% of the amebas remain). We calculated ratios of the number of lysis plaques obtained with respect to that for the control *Klebsiella aerogenes*. Data are mean values from three independent experiments (the standard deviation is shown).

We carried out infection tests with eukaryotic animal and plant cell models. MFE01 cytotoxicity toward glial cells was quantified by measuring lactate dehydrogenase (LDH) release into the culture medium. No significant cytotoxicity was observed after 4 h or 24 h in the MFE01/glial cell contact assay, indicating that MFE01 is avirulent against glial cells (Fig. 2A). The chicory leaf soft-rot test was used to assess MFE01 virulence against plant cells. *P. aeruginosa* H103, the positive control strain for virulence, caused soft rot on chicory leaf. MgSO_4_, the negative control, and MFE01 did not induce symptoms on chicory leaf. We can therefore conclude that MFE01 is avirulent toward this plant cell model (Fig. 2B), consistent with the beneficial nature of many strains of this species to plants [45].

We then assessed the ability of *P. fluorescens* MFE01 to resist phagocytic predation by the ameba *Dictyostelium discoideum*. On a layer of *Klebsiella aerogenes* [46] containing 10% MFE01, about a hundred lytic plaques corresponding to zones in which amebas were feeding on bacteria were observed. This suggests that MFE01 does not protect against predation by amebas (Fig. 2C). This absence of virulence in these models is consistent with the avirulent phenotype of the saprophytic members of this species, but not with the results obtained for MFN1032, a clinical isolate of *Pseudomonas fluorescens* that can also grow at 37°C [38].

**Figure 3:**
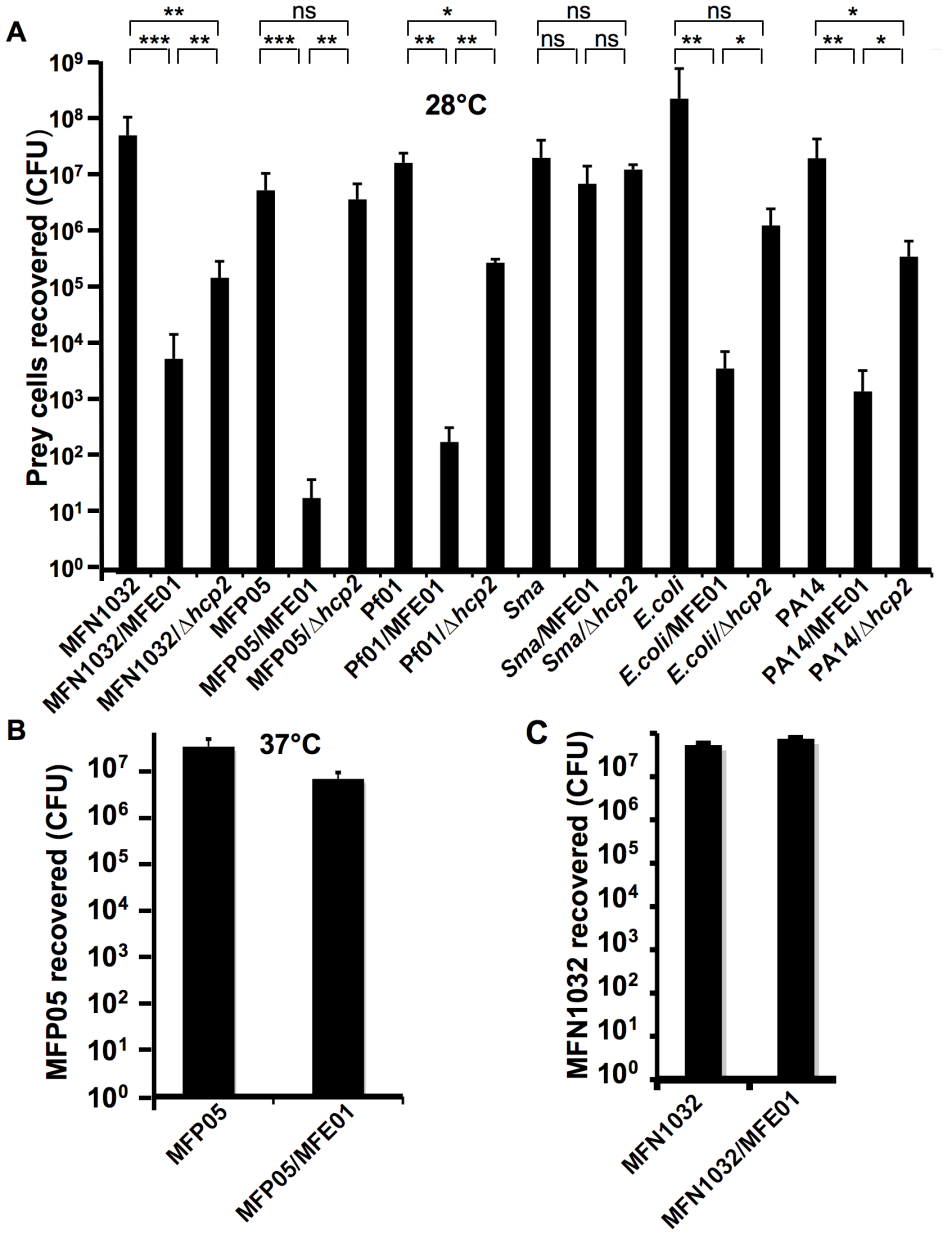
*P. fluorescens* MFE01 displays bacterial killing activity. For each assay, *N* = 4, and the error bars represent the standard error of the mean. (**A**) A quantitative coculture assay was performed for 4 h at 28°C. Different prey cells were incubated with or without *P. fluorescens* MFE01 or MFE01*Δhcp2 (Δhcp2)*, at a ratio of 1∶5, respectively. Statistics were done by pairwise strain comparisons (non-parametric Mann-Whitney-two tailed Test): *p-value <0.05, **p-value <0.02, ***p-value <0.002, ns no significant difference. *Sma : Serratia marcescens.* (**B**) *Pseudomonas fluorescens* MFP05 was incubated with or without wild-type MFE01 (ratio 1∶5) for 4 h at 37°C for coculture assays. (**C**) Quantitative assessment of the MFN1032 population cocultured for 4 h at 28°C with MFE01 or without MFE01. A filter with 0.22 µm pores was placed between MFE01 and MFN1032, to prevent physical contact.

Given this lack of evidence for MFE01 virulence against various eukaryotic cell models (amebas, plant and glial cells), coupled with several reports of T6SS-dependent antibacterial activity, we investigated whether *P. fluorescens* MFE01 had antibacterial activity. Antibacterial competition assay consists to mix MFE01 and prey bacteria at a ratio of 5∶1, respectively, and co-cultured on an agar plate for 4 h. We then counted the number of surviving prey cells. At 28°C, prey bacteria which co-cultivated with MFE01 exhibited a drastic and significant population drop, from three- log lost for *Pseudomonas fluorescens* MFN1032, four-log lost for *P. aeruginosa* PA14, five-log for *E. coli* or *Pseudomonas fluorescens* Pf01 and six–log drop for *P. fluorescens* MFP05 compared to results for cultures without MFE01 (Fig. 3A). *Serratia marcescens* was the only bacterium tested that was resistant to predation by MFE01 at 28°C (Fig. 3A). We concluded that MFE01 had a bacterial killing activity at 28°C.

We investigated whether this killing activity was linked to Hcp secretion, by monitoring antibacterial activity at 37°C (Fig. 3B). *P. fluorescens* MFP05 did not die during coculture at 37°C, while it was the strain most sensitive to MFE01 predation at 28°C. We investigated whether MFE01 killing activity was contact-dependent. A new antibacterial competition assay was carried out at 28°C, without contact between the predator MFE01 and the prey MFN1032 (Fig. 3C). The competitor MFN1032 survived if it was separated from MFE01 by a filter with 0.22 µm pores. In these conditions, no decrease was observed in the recovery of viable *P. fluorescens* MFN1032. MFE01 appeared to lose its killing activity in the absence of close contact. In other species, the antibacterial effect mediated by the T6SS has been shown to be contact-dependent [47], and competition can be abolished by a filter separating the predator and prey, suggesting that MFE01 T6SS is involved in *P. fluorescens* competition.

### The MFE01 T6SS is Involved in Predation on Various Gram-negative Bacteria

A MFE01 genomic bank was established for analysis of the genomic environment of the *hcp2* gene. Sequencing revealed that there was a *vgrG* gene located downstream from *hcp2*. A MFE01Δ*hcp2* mutant was constructed by introducing an early stop codon in the middle of the *hcp2* gene. Wild-type MFE01 and MFE01Δ*hcp2* mutant growth curves were similar, indicating that *hcp2* mutation had no effect on growth kinetics.

**Figure 4:**
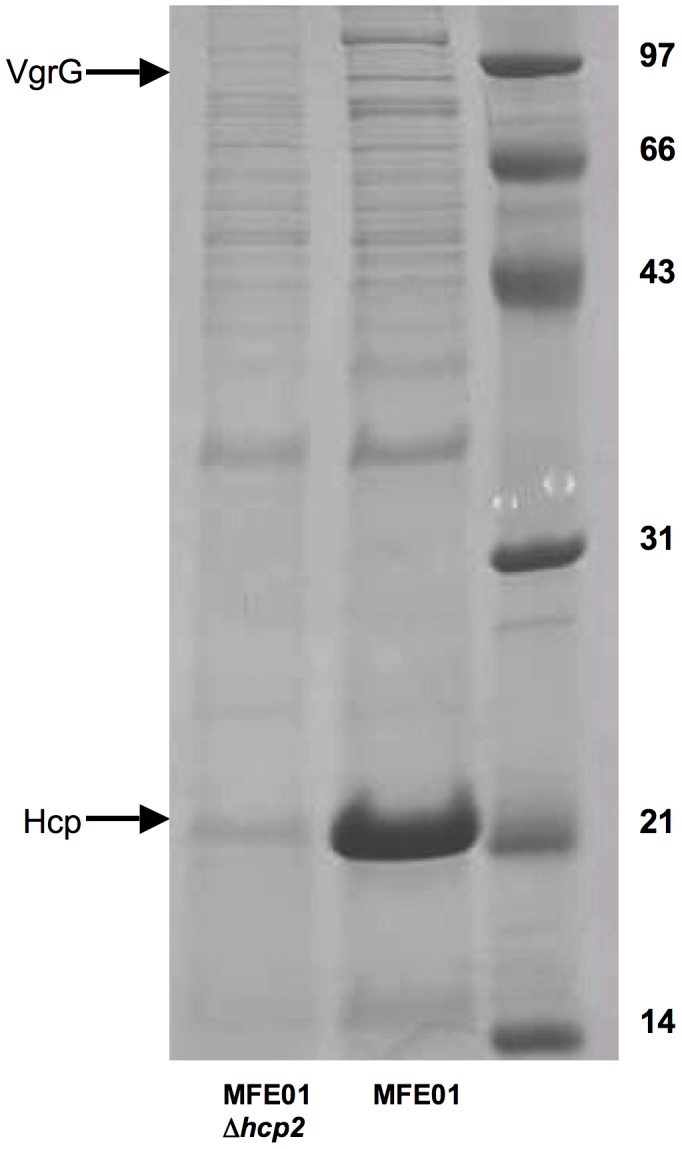
*hcp2* mutation inhibits VgrG secretion. Concentrated culture supernatants for wild-type MFE01 and mutant MFE01Δ*hcp2* were analyzed by SDS-PAGE and Coomassie Blue staining, for bacteria in stationary growth phase after incubation at 28°C. MFE01Δ*hcp2* displayed much lower levels of Hcp secretion (indicated by an arrow) than MFE01. The residual band was analysed by mass spectroscopy and identified as an Hcp-like protein. The second arrow indicates the absence of the band corresponding to the VgrG-like protein in MFE01Δ*hcp2* supernatant.

However, *hcp2* mutation resulted in much lower levels of Hcp protein secretion into the medium than for wild-type MFE01. However, a weak band corresponding to a Hcp-like protein (identified by mass spectrometry) was still visible on the MFE01Δ*hcp2* strip (matched with gi:398853281, hcp1 family from *Pseudomonas* Sp. GM 80, score of 108, coverage of 40%, match with 8 peptides) (Fig. 4). This residual Hcp secretion may reflect *hcp1* expression. The *hcp2* gene seems to be responsible for most of the observed Hcp-like proteins secretion. Mougous and coworkers have reported considerable heterogeneity in Hcp secretion within *P. aeruginosa,* depending on the *hcp* gene concerned [2], [48].

We investigated the possible role of the *hcp2* gene in MFE01 killing activity, by performing competition assays at 28°C with MFE01Δ*hcp*2 (Fig. 3A). During competition with PA14, MFN1032 and Pf01, MFE01Δ*hcp2* reduced significantly prey cell population, but at a significantly lower level than MFE01.

In MFP05 and *E.coli* competition assay, killing activity was abolished in MFE01Δ*hcp2,* which had no significant effect on the prey cell population (Fig. 3A). Similar findings have also been reported for the T6SSs of *V. cholerae* [18], *A. baumannii* [14], *B. thailandensis* [16] and *P. aeruginosa* (HSI-I) [17], consistent with a role for the *P. fluorescens* T6SS in bacterial competition. According to the prey cell, this T6SS seems to act or not in synergy with another unknown mechanism.

We introduced pPSV35 [49], carrying native *hcp2,* into MFE01Δ*hcp2,* to obtain MFE01Δ*hcp2/hcp2.* The expression of this wild-type *hcp2* gene in *trans* did not restore the wild-type phenotype (data not shown). Carruthers and coworkers encountered the same problem with *Acinetobacter baumannii* and suggested that it probably resulted from polar effects on downstream genes in the T6SS cluster [14]. Polar effects may be responsible for complementation failure if the *vgrG* and *hcp2* genes are cotranscribed. We tested this hypothesis, by using the hcp-vgrgF and hcp-vgrgR primers, corresponding to the *hcp2* and *vgrG* regions, respectively, for PCR on cDNA from MFE01 growing exponentially at 28°C. No amplification was detected with these primers, whereas *hcp2* was expressed, suggesting that *hcp2* and *vgrG* were not cotranscribed (data not shown). To confirm this result, we used vgrG primers, to quantify transcription *of vgrG* in MFE01 and MFE01Δ*hcp2.* We observed no significant difference of *vgrG* mRNA level between these two strains. We then hypothesized that MFE01Δ*hcp2* might have another mutation outside the *hcp2* gene. We checked for the absence of such a mutation in MFE01Δ*hcp2*, by reintroducing the native *hcp2* gene at its usual chromosomal location to obtain the revertant strain MFE01Δ*hcp2*-R. Coculture assays indicate that MFE01Δ*hcp2*-R recovered killing activity, demonstrating that an unidentified mutation was not responsible for complementation failure. We suggest that fine regulation of the dynamics of this overexpressed T6SS occurs during the association between the various constitutive proteins of the T6SS apparatus (Hcp, VgrG, VipA,VipB) and that this regulation is disturbed during the expression of the *hcp2* gene in *trans*.

MFE01 seems to have a large target cell spectrum. Basler and coworkers have shown that the T6SS of prey cells may affect the activation of the predator T6SS in *Pseudomonas aeruginosa* [50]. We found that expression of a T6SS in Pf01, or no expression in MFP05, had no significant impact on the potential activation of killing activity in MFE01. We showed that the *hcp* genes of MFE01 were similar to Pf01 *hcp* genes, but Pf01 was unable to resist MFE01 predation. This suggests that MFE01 is capable of antagonizing Pf01 by variation in effector and immunity proteins according to recent works [26], [51].

### T6SS Immunity Proteins of *S. marcescens* Protect *E. coli* Against MFE01

**Figure 5:**
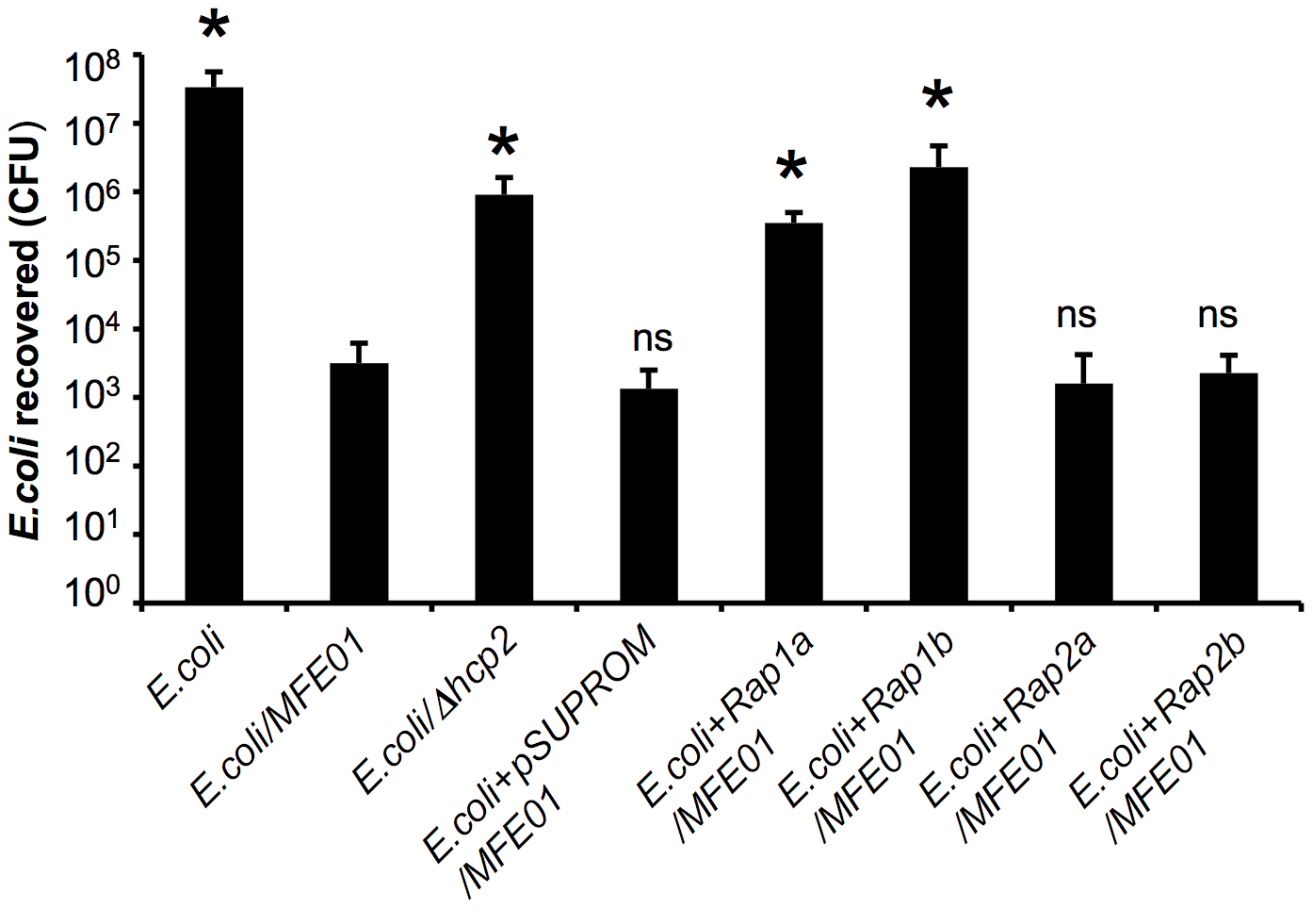
Rap immunity proteins from *Serratia marcescens* protect *E. coli* against MFE01. Recovery of viable *E. coli* cells harboring a pSUPROM vector conferring the constitutive production of various *Serratia marcescens* immunity proteins, Rap 1a, 1b, 2a or 2b, or an empty pSUPROM vector, as control. Cocultures were performed between the indicated *E. coli* strains and wild-type *P. fluorescens* MFE01 or MFE01*Δhcp2* (*Δhcp2*) (ratio 1∶5, respectively) at 28°C (*N* = 4 for each assay). * Indicates a significant difference in *E.coli* CFU (*p*-value <0.05) when compared to the *E.coli*/MFE0 assay. ns means no significant difference.

For confirmation of the role of the T6SS in bacterial killing activity, we made use of the resistance of *S. marcescens* to MFE01. We investigated whether the Rap immunity proteins were responsible for protecting *S. marcescens* against MFE01, explaining the number of viable *S. marcescens* cells recovered (Fig. 3A). We used an approach similar to that used by English and coworkers to demonstrate cross-immunity between two effector-immunity (EI) protein families by the introduction of Rap immunity proteins into *E. coli* [20]. Fig. 5 shows *E. coli* harboring the pSUPROM vector constitutively expressing the Rap proteins indicated in coculture with wild-type *P. fluorescens* MFE01.

The expression of Rap1a and Rap1b increased the counts of viable *E. coli* by two-log with respect to the counts obtained for *E. coli* with empty vector. However, Rap2a and Rap2b conferred a resistance similar to that obtained with the empty vector. Although killing activity was only partially suppressed, this experimental approach highlighted the existence of cross-immunity, providing support for a role of *hcp2* in MFE01 killing activity mediated by the effectors of T6SS. *E. coli* does not express all the Rap immunity proteins, accounting for the absence of full protection resembling that observed for *S. marcescens*. According to nomenclature of Russell *et al.*, the *S. marcescens* toxins Ssp1 and Ssp2 belong to the Tae4 protein family, and are responsible for hydrolyzing peptide crosslinks at the γ-D-glutamyl-*m*DAP DL-bond [25]. These findings suggest that MFE01 secretes toxins targeting the peptidoglycan of Gram-negative bacteria.

### MFE01 Inhibits the Growth of *Pectobacterium atrosepticum* 6276

**Figure 6:**
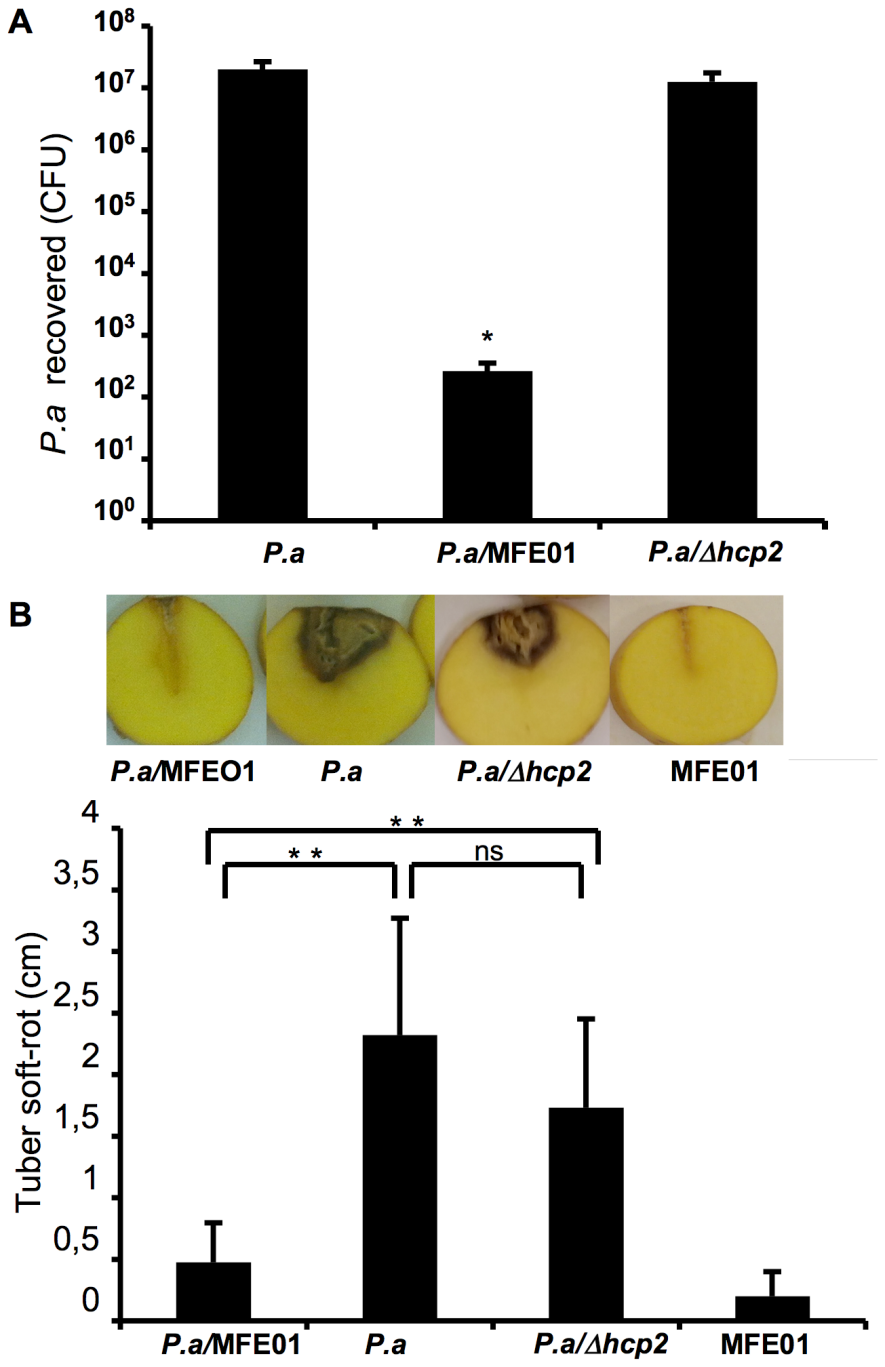
MFE01 protects against tuber soft-rot. This figure shows a soft-rot assay. (**A**) Coculture assay for MFE01 or MFE01*Δhcp2* (*Δhcp2*) and *Pectobacterium atrosepticum* (*P.a*), for 4 h at 28°C (ratio 5∶1, respectively) (*N* = 4 for each assay). * Indicates a significant difference in *P.a* CFU (*p*-value <0.05) when compared to the *P.a* control. (**B**) *Pectobacterium atrosepticum* (*P.a*) was incubated with or without *P. fluorescens* MFE01 or MFE01*Δhcp2* (*Δhcp2*) at a ratio of 1∶10, respectively. Each potato tuber was inoculated and incubated for 7 days at 25°C. Soft-rot diameter was monitored. The error bars indicate the standard error of the mean and the images shown are representative. Statistics were done by pairwise strain comparisons (non-parametric Mann-Whitney-two tailed Test): **p-value <0.01, ns no significant difference.

As *Pseudomonas fluorescens* has been described as a major PGPR (plant growth-promoting rhizobacterium) [52], we assessed the killing activity of MFE01 against *Pectobacterium atrosepticum* 6276, which causes tuber soft rot. MFE01 killed *P. atrosepticum in vitro* (Fig. 6A), decreasing cell counts by five-log whereas MFE01Δ*hcp2* had no effect on *Pectobacterium atrosepticum.* We assessed the ability of the predator activity of MFE01 to protect tubers against *P. atrosepticum in planta*. MFE01 or MFE01Δ*hcp2* were mixed with *P. atrosepticum* in a 10∶1 ratio and the mixture obtained was then used to inoculate tubers. MFE01 protection was evaluated by determining the presence or absence of tuber soft rot. MFE01 was not pathogenic to tubers and it significantly inhibited the development of soft rot due to *P. atrosepticum.* Conversely, MFE01Δ*hcp2* did not protect tubers against *P. atrosepticum* (Fig. 6B). This result suggests that the T6SS of MFE01 may be involved in this tuber protection.

## Conclusion

We describe here the first example of a functional T6SS in the environmental *P. fluorescens* strain MFE01. We obtained strong evidence that *P. fluorescens* MFE01 T6SS plays an important role in competition with other bacteria. It is easy to see how this competitive ability would help the bacterium to survive and to prosper in the environmental reservoir or to compete in a new niche. The unusual constitutive production and over-secretion of Hcp in this strain indicates that this T6SS is very important for the fitness of MFE01. The high level of Hcp secretion in this strain suggests that the benefits of this overproduction are greater that the energetic cost, again highlighting the importance of this system. An understanding of this mechanism would facilitate the development of new methods of combating bacterial proliferation, making use of the inhibitory properties reported for the MFE01 strain.
